# Estimated glucose disposal rate and risk of cardiovascular disease: evidence from the China Health and Retirement Longitudinal Study

**DOI:** 10.1186/s12877-022-03689-x

**Published:** 2022-12-15

**Authors:** Xiao Ren, Minglan Jiang, Longyang Han, Xiaowei Zheng

**Affiliations:** Public Health Research Center and Department of Public Health and Preventive Medicine, Wuxi School of Medicine, Jiangnan University, 1800 Lihu Road, Binhu District, Jiangsu Province 214122 Wuxi, China

**Keywords:** Estimated glucose disposal rate, eGDR, CVD, CHARLS

## Abstract

**Objectives:**

Previous studies had reported that insulin resistance (assessed by estimated glucose disposal rate; eGDR) was associated with higher risk of cardiovascular events (CVD) in diabetes patients. The aim of present study was to investigate the potential association between eGDR and CVD in general population.

**Methods:**

The China Health and Retirement Longitudinal Study with 8,267 individuals were included in analysis. Participants were divided into four subgroups according to eGDR quartile. Cox proportional hazards regression models were used to examine the associations of eGDR with CVD (stroke or cardiac events).

**Results:**

During 6 years of follow-up, a total of 1,476 respondents experienced a CVD (494 stroke and 1,110 cardiac events). In multivariable-adjusted analyses, the corresponding hazard ratio *(95% confidence intervals*) for the highest eGDR versus lowest quartile of eGDR was 0.58(0.49–0.67) for CVD. Each 1-SD increase of eGDR was associated with 16% (HRs = 0.84; 0.79–0.88) decreased risk of CVD. There was also a significant linear association between eGDR and CVD (*P* for linearity < 0.001). Similar associations were also found between eGDR and stroke and cardiac events.

**Conclusion:**

A higher eGDR (a measure of insulin resistance) was associated with a decreased risk of CVD, stroke and cardiac events in general Chinese population, suggesting that eGDR could be considered as a preferential predictor and treatment target of CVD. Future well-designed prospective clinical studies are needed to verify our findings and to assess the effect of eGDR interventions in CVD prevention and therapy.

**Supplementary Information:**

The online version contains supplementary material available at 10.1186/s12877-022-03689-x.

## Introduction

Cardiovascular disease (CVD) is still the leading cause of mortality worldwide and responsible for 18.6 million deaths in 2019, which was estimated to account for 32.3% of all-cause global deaths [1–3]. Atherosclerosis is the major cause of CVD and stroke [4]. Insulin resistance and the ensuing hyperinsulinemia/hyperglycemia are crucial link between atherosclerosis and CVD [5–7]. Previous studies reported that preventing insulin resistance would prevent almost 40% of atherosclerotic disease regardless of hypertension, hyperlipidemia, hyperglycemia or obesity, involved in the insulin resistance state [8].

In clinic, the gold standard technique measuring insulin resistance is the euglycaemic hyperinsulinaemic clamp method [9], while the homeostasis model assessment of insulin resistance (HOMA-IR) is widely used as a valid surrogate of the gold standard [10]. However, the above detection methods were invasive and costly, and therefore not suitable for large-scale daily clinical use. In recent years, a validated score against the euglycaemic hyperinsulinaemic clamp based on the readily available clinical factors waist circumference, hypertension, and glycosylated hemoglobin A1c (HbA1c) was developed to estimate the glucose disposal rate (eGDR) in patients with type 1 diabetes and type 2 diabetes, which has been proven to have a high precision when compared to the euglycaemic hyperinsulinaemic clamp method [10, 11]. Several previous studies had investigated the utility of eGDR and results indicated this metric was significant associated with preclinical carotid atherosclerosis [10], coronary artery disease [12], stroke [13] and mortality [14] in individuals with type 1 diabetes (T1D) or type 2 diabetes (T2D). Evidence from a cross-sectional study also indicated that eGDR could improve the identification of prevalent ischemic heart disease in the Chinese rural general population [15]. However, whether grade of insulin resistance predicts CVD in general population is not well known.

In current study, we aimed to evaluate the association between insulin resistance (IR, assessed by eGDR) and risk of CVD (stroke or cardiac events) among middle aged and older Chinese adults based on the data from the China Health and Retirement Longitudinal Study (CHARLS).

## Methods

### Study population

CHARLS is an ongoing nationally representative and population-based study, that uses a multistage clustering sample method to select participants and conducted to collect a series of data regarding sociodemographic and lifestyle factors and health-related information in Chinese citizens aged 45 years or older [16, 17]. In CHARLS 2011, a total of 17,708 participants in 10,257 households were recruited from 150 counties or districts and 450 villages within 28 provinces in China. All participants were followed up every 2 years after the baseline survey [18]. The ethics application for collecting data on human subjects in CHARLS was approved by the Biomedical Ethics Review Committee of Peking University (IRB00001052-11,015), and performed in accordance with s the Declaration of Helsinki. Furthermore, all CHARLS participants provided written informed consent. The details of the CHARLS data are available at its website (http://charls.pku.edu.cn/en).

**Fig. 1:**
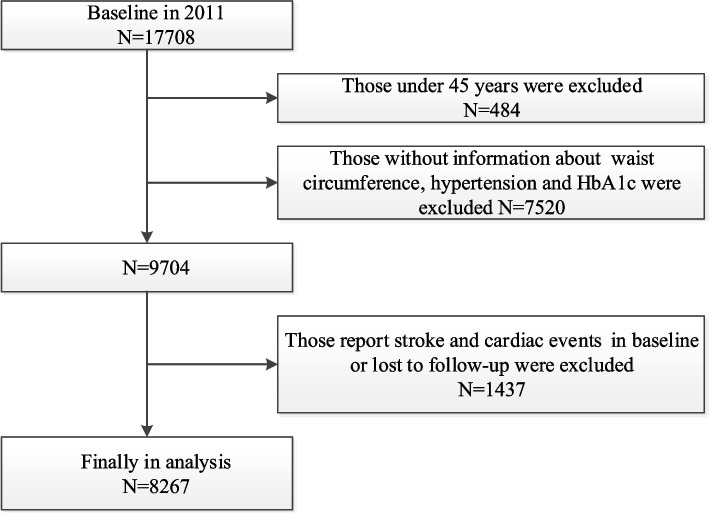
Flow chart of sample selection and the exclusion

In current study, a total of 17,708 individuals were included in the baseline survey of around 450 basic communities from 28 provinces in China. The exclusion criteria were as follows: 1) individuals < 45 years old (*N* = 484); 2) individuals without complete information about waist circumference, hypertension and Hemoglobin A1c (HbA1c) (*N* = 7520); 3) individuals who reported stroke and cardiac events in baseline or lost to followed-up (*N* = 1437). Finally, a total of 8,267 individuals were eligible for subsequent analysis (Fig. 1).

### Blood sample collection and measurement of HbA1c

Every participant was demanded to fast overnight, and their blood samples were collected by medically trained staff. Then the samples were transported to the local laboratory timely and stored at 4 °C. The blood samples were centrifugated and stored at -20 °C before been transported to the central laboratory in Beijing and frozen at—80 °C before analysis [19]. All study laboratories had the standardized certification. The fasting plasma glucose (FPG) concentrations were measured using an enzymatic colorimetric test method, whereas the HbA1c assay was performed using the boronate affinity high performance liquid chromatography method [19].

### Estimated glucose disposal rate measurement and outcome assessments

In current study, the eGDR (mg/kg/min) was calculated as previously described according to the following formula: eGDR = 21.158-(0.09 * WC) -(3.407 * HT) -(0.551* HbA1c) [WC = waist circumference (cm), HT = hypertension (yes = 1/no = 0), and HbA1c = HbA1c (%DCCT)] [20].

The primary outcome in current study was new-onset CVD (stroke or cardiac events), and the secondary outcomes were new-onset stroke and cardiac events, separately. The new-onset stroke or cardiac events were assessed by the following questions: “Have you been told by a doctor that you have been diagnosed with a heart attack, angina, coronary heart disease, heart failure, or other heart problems?” or “Have you been told by a doctor that you have been diagnosed with a stroke?” [21, 22]. Special conditions were employed to improve the accuracy of estimation of the onset time of stroke, as the exact time of stroke development was not available for all participants. They were considered as follows: First, if the participants did not develop stroke in any of the follow-up wave surveys (the time to event was calculated as follows: the time of the last survey—the time of baseline investigation); the follow-up time was not available (the approximate estimated time to event is defined as follows: the integer number years of the time of the last survey—the time of baseline investigation). Second, if they developed stroke (the time to event was defined as: the time of specific wave with stroke information/2—the time of interval wave/2 + the time of interval wave—the time of baseline investigation); the follow-up time was not available (the approximate time to event is defined as the integer number years of the time of specific wave with stroke information/2—the time of interval wave/2 + the time of interval wave—the time of baseline investigation). The same calculation is also used for cardiac events.

### Covariates assessments

The covariates were collected at baseline including age, sex, place of residence (rural vs. urban), smoking status (ever smoking vs. never smoking), educational level (illiteracy; primary school; middle school; high school or above), drinking status (ever drinking vs. never drinking), body mass index (BMI; the weight in kilograms divided by the square of the height in meters), the presence or absence of other chronic diseases (dyslipidemia, chronic lung disease) and medications (anti-hypertensive and anti-dyslipidemic). Diabetes was defined as fasting glucose 126 mg/dl, or glycosylated hemoglobin (HbA1c) 6.5%, or treatment for diabetes mellitus, or self-reported history of diabetes. Dyslipidemia was defined as triglycerides 150 mg/dl, TC 240 mg/dl, HDL-C < 40 mg/dl, LDL-C 160 mg/dl, current use of the lipid-lowering medications, or self-reported history of dyslipidemia. Respondents were defined as having doctor-diagnosed chronic lung diseases if they answered yes to the question (“Have you been diagnosed with chronic lung diseases (excluding tumors, or cancer)”). The definition of smoking, drinking and hypertension has been described in previous study [18].

### Statistical analysis

Participants were divided into four subgroups according to eGDR (mg/kg/min) quartiles. Data was given in the form of means ± standard deviations (SD) for continuous variables of the normal distribution or as median (interquartile range) for continuous variables of the abnormal distribution, and as frequency (percentage) for categorical variables. Pearson’s χ^2^ test was performed to compare the distribution of categorical variables, and ANOVA or Kruskal–Wallis test was performed to compare the continuous variables. Cox proportional hazards model was used to calculate the hazard ratios (HRs) and 95% confidence intervals (CIs) for new-onset CVD, stroke and cardiac events before and after adjusting for covariates. Kaplan–Meier curves and the log-rank test were used to compare the cumulative risk of CVD, stroke and cardiac events among four subgroups. In multivariable-adjusted model, both age, sex, place of residence, education level, smoking, drinking, systolic blood pressure, fasting plasma glucose, physical activity, chronic diseases (dyslipidemia, chronic lung disease) and medications (anti-hypertensive and anti-dyslipidemic) were included in the multivariable models. Furthermore, restricted cubic splines were used to examine the shape of the association between eGDR and CVD, stroke and cardiac events with four knots (at the 5th, 35th, 65th, and 95th percentiles) [23].

Subgroup analyses were further performed to evaluate the association between eGDR levels and the risk of CVD according to sex, diabetes, age, place of residence, smoking, drinking, education level and anti-diabetes/hypertension drugs subgroups. In sensitivity analysis 1, we calculated eGDR using BMI instead of waist circumferences. The eGDR_BMI_ based on BMI was calculated according to the following formula: eGDR_BMI_ = 19.02- (0.22* BMI)- (3.26*HT)- (0.61*HbA1c) (BMI = body mass index(kg/m2), HT = hypertension (yes = 1/no = 0), and HbA1c = HbA1c (%)) [13]. Two tailed *P* < 0.05 was considered to be statistically significant. In sensitivity analysis 2, those with anti-diabetes/hypertension drugs use were excluded in analysis. In sensitivity analysis 3, we further evaluate the effect of longitudinal changes of eGDR on CVD, stroke and cardiac events. Change in eGDR over time was calculated as the difference in eGDR between wave 1 and wave 3. All statistical analyses were conducted using SAS statistical software (version 9.4, Cary, NC).

## Results

In the current study, a total of 8,267 participants (3,916 men and 4,351 women) were included in the analysis, and the average age was 58.90 ± 9.40 years. The median value of eGDR was 10.41(8.92–11.26) mg/kg/min. Baseline characteristics between the included and excluded participants were shown in Supplemental Table 1. As shown in Table 1, baseline characteristics, such as age, living place, education level, dyslipidemia, smoking, blood glucose, BMI, SBP and DBP were significantly different among the four subgroups.

**Table 1:** Baseline characteristics of the study participants according to eGDR quartiles

Characteristics	eGDR(mg/kg/min)				*P* value
	< 8.92	8.92–10.41	10.41–11.26	≥ 11.26	
No. of subjects	2067	2072	2071	2057	
Age, years	59.99 ± 8.71	58.43 ± 9.39	58.26 ± 9.49	58.91 ± 9.87	< 0.001
Sex, n (%)					
Male	1053(50.94)	865(41.75)	1006(48.58)	992(48.23)	0.788
Female	1014(49.06)	1207(58.25)	1065(51.42)	1065(51.77)	
Living place, n (%)					
Urban	788(38.12)	789(38.08)	633(30.56)	552(26.84)	< .0001
Rural	1279(61.88)	1283(61.92)	1438(69.44)	1505(73.16)	
Education level, n (%)					
Below primary school	589(28.50)	625(30.16)	618(29.84)	676(32.86)	< 0.001
Primary school	862(41.70)	793(38.27)	849(38.27)	849(40.99)	
Middle school	417(20.17)	431(19.28)	425(20.52)	425(20.52)	
High school or above	199(9.63)	223(10.76)	179(8.64)	179(8.64)	
Dyslipidemia, n (%)	242(11.71)	186(8.98)	124(5.99)	71(3.45)	< 0.001
Smoking, n (%)	848(41.03)	705(34.03)	840(40.56)	893(43.41)	0.005
Drinking, n (%)	862(41.70)	785(37.89)	806(38.92)	824(40.06)	0.417
Blood glucose, mg/dl	120.57 ± 55.47	112.22 ± 33.52	104.46 ± 25.07	102.30 ± 11.73	< 0.001
BMI, kg/m^2^	23.96(21.52–27.03)	25.60(23.83–27.46)	22.67(21.20–24.12)	20.32(18.87–21.88)	< 0.001
SBP, mmHg	131.66 ± 20.81	133.36 ± 21.56	128.98 ± 21.13	125.43 ± 20.28	< 0.001
DBP, mmHg	76.16 ± 11.95	77.75 ± 11.77	75.398 ± 11.83	73.08 ± 11.17	< 0.001

Continuous variables are expressed as mean ± standard deviation, or as median (interquartile range). Categorical variables are expressed as frequency (percent)

*BMI* Body mass index, *SBP* Systolic blood pressure, *DBP* Diastolic blood pressure

**Fig. 2:**
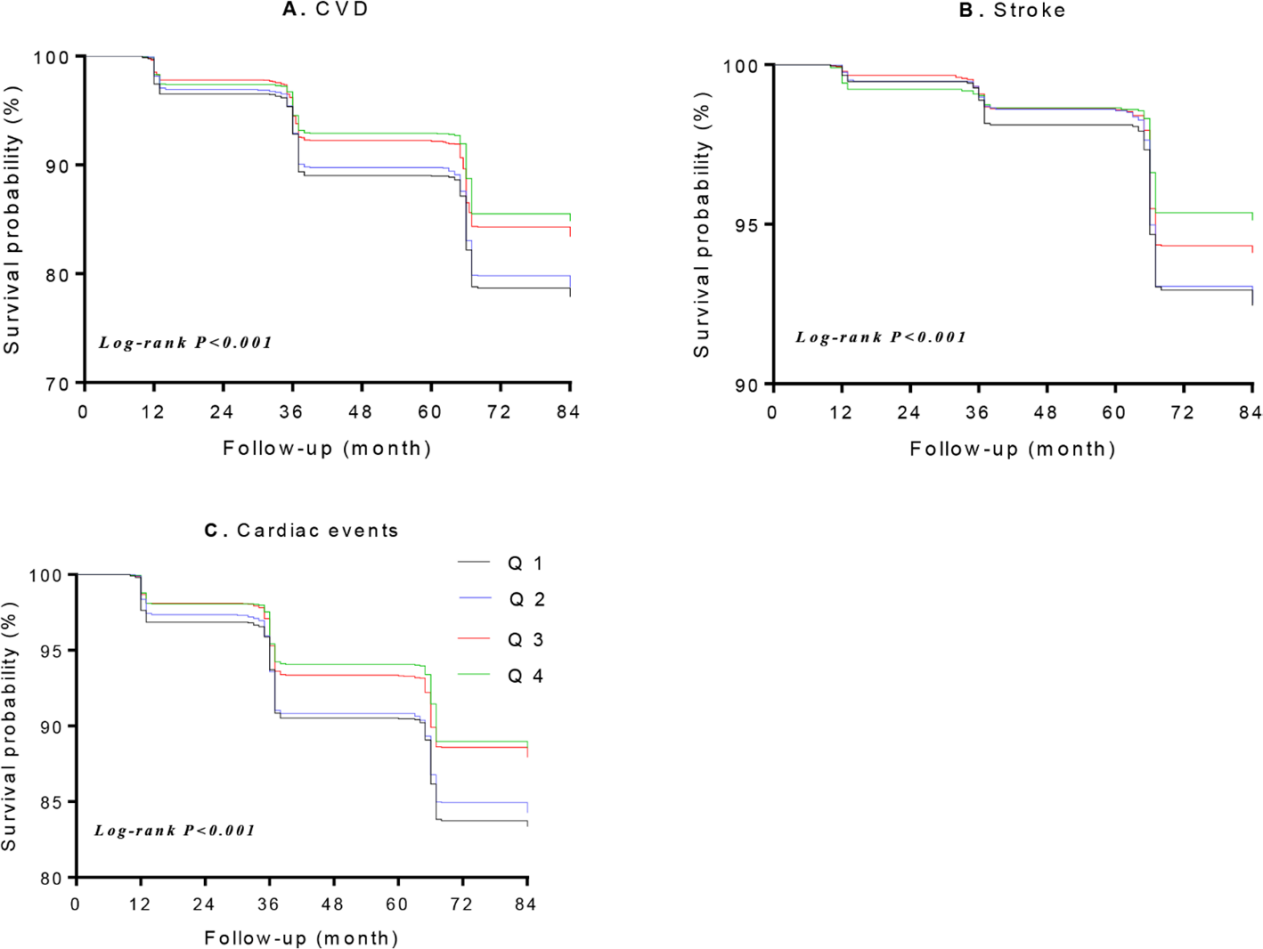
Survival curves according to eGDR quartiles for (**A**)CVD; (**B**) stroke; (**C**) cardiac events

**Fig. 3:**
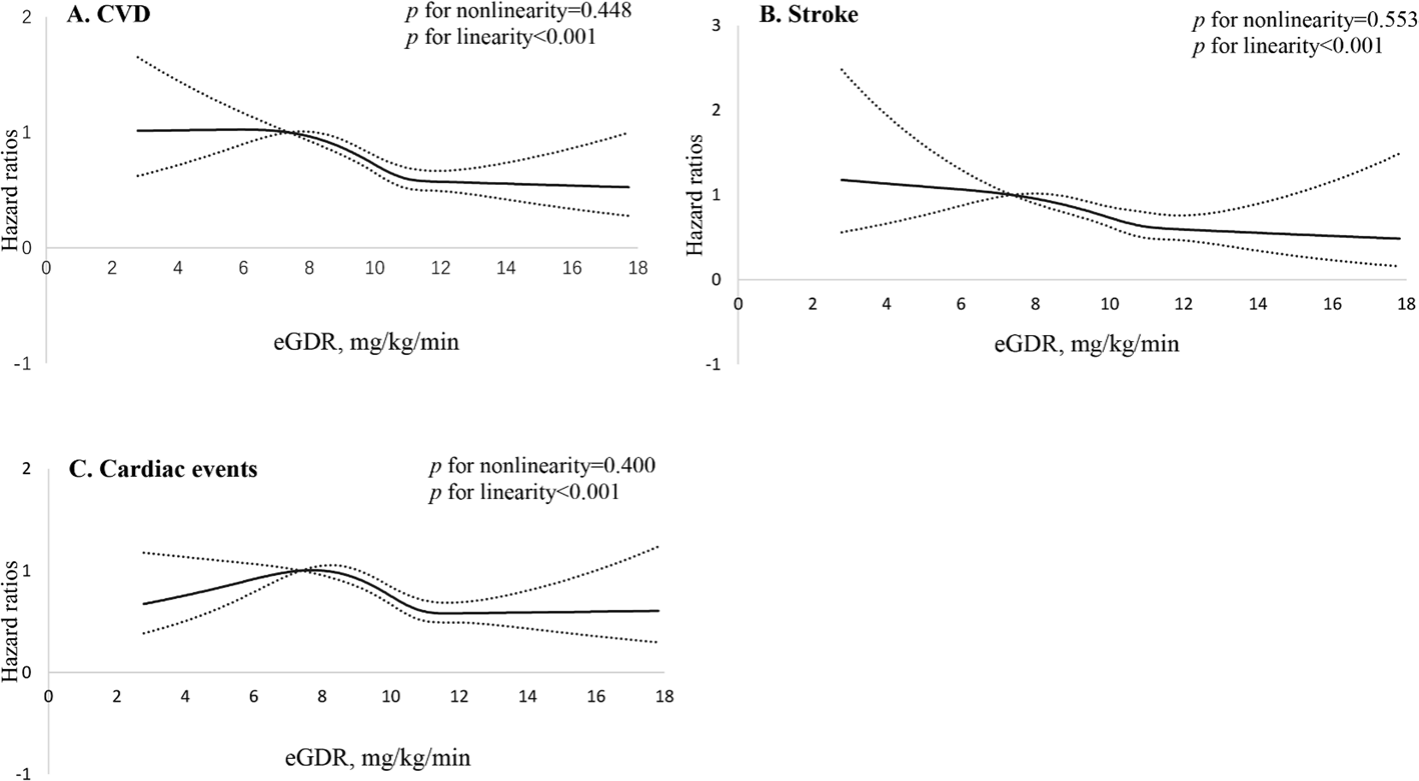
Association of eGDR quartlies with risk of CVD, stroke and cardiac events. Hazard ratios and 95% CIs derived from restricted cubic spline regression, with knots placed at the 5th, 35th, 65th, and 95th percentiles of the distribution of remnant cholesterol. The reference point for remnant cholesterol is the midpoint (7.38 mg/kg/min) of the reference group from categorical analysis. The lowest 1% and highest 1% of participants were not shown in the figures for small sample sizes. Hazard ratios were adjusted for the same variables as multivariable-adjusted model in Table 2

After 6 years of follow-up (Wave 2 to Wave 4), a total of 1,476 respondents experienced (494 stroke and 1110 cardiac events). Event rates for CVD, stroke and cardiac events are shown in Table 2. Kaplan–Meier analysis showed that participants in the top quartile of eGDR had significantly lower cumulative incidence rates of CVD, stroke and cardiac events (all log-rank *P* < 0.001; Fig. 2). After adjustment for age, sex and other variables, the HRs (*95%CIs*) for the highest quartile of eGDR was 0.58(0.50–0.67) for CVD, 0.66(0.50–0.88) for stroke and 0.58(0.48–0.69) for cardiac events, respectively, compared with the lowest quartile. Moreover, each 1-SD increase of eGDR was associated with 19% (*95%CIs,* 11%-23%), 12%(*95%CIs*, 3%-19%) and 16%(*95%CIs,* 11%-20%) decreased risk of CVD, stroke and cardiac events, respectively (Table 2). Multivariable-adjusted restricted cubic spline analyses presented linear associations between eGDR with CVD, stroke and cardiac events (all *P* for linearity < 0.001) (Fig. 3).

**Table 2:** Event rates and relative risks for CVD, stroke and cardiac events by eGDR quartlies

Characteristics	eGDR(mg/kg/min)				*P* trend	Each SD increase^a^
	< 8.92	8.92–10.41	10.41–11.26	≥ 11.26		
**CVD**						
Case, n(%)	457(22.11)	440(21.24)	314(15.16)	265(12.88)		
Unadjusted	1.00(Ref)	0.96(0.84–1.09)	0.66(0.57–0.76)	0.56(0.48–0.65)	< 0.001	0.82(0.78–0.86)
Age and sex- adjusted	1.00(Ref)	0.96(0.84–1.09)	0.67(0.58–0.78)	0.56(0.48–0.65)	< 0.001	0.83(0.79–0.87)
Multivariable-adjusted^b^	1.00(Ref)	0.95(0.83–1.10)	0.69(0.59–0.80)	0.58(0.49–0.67)	< 0.001	0.84(0.79–0.88)
**Stroke**						
Case, n(%)	156(7.55)	155(7.48)	101(4.88)	82(3.99)		
Unadjusted	1.00(Ref)	0.99(0.79–1.23)	0.64(0.50–0.82)	0.52(0.40–0.68)	< 0.001	0.80(0.74–0.87)
Age and sex- adjusted	1.00(Ref)	1.04(0.83–1.30)	0.67(0.52–0.86)	0.53(0.41–0.70)	0.002	0.81(0.75–0.88)
Multivariable-adjusted^b^	1.00(Ref)	1.01(0.81–1.27)	0.76(0.58–0.99)	0.70(0.53–0.94)	0.004	0.91(0.83–0.99)
**Cardiac events**						
Case, n(%)	344(16.64)	326(15.73)	237(11.44)	203(9.87)		
Unadjusted	1.00(Ref)	0.94(0.81–1.10)	0.67(0.57–0.79)	0.57(0.48–0.68)	< 0.001	0.84(0.79–0.89)
Age and sex- adjusted	1.00(Ref)	0.93(0.80–1.08)	0.68(0.57–0.80)	0.58(0.48–0.68)	< 0.001	0.84(0.79–0.89)
Multivariable-adjusted^b^	1.00(Ref)	0.94(0.81–1.09)	0.68(0.58–0.81)	0.57(0.47–0.68)	< 0.001	0.84(0.79–0.89)

*CVD* Cardiovascular diseases, *eGDR* Estimated glucose disposal rate

^a^Each SD increase in eGDR (1.71 mg/kg/min)

^b^Multivariable-adjusted for age, sex, place of residence, education level, blood glucose, smoking, drinking, systolic blood pressure, physical activity, chronic diseases (dyslipidemia, chronic lung disease) and medications (anti-hypertensive and anti-dyslipidemic)

In the subgroup analysis, significant associations between eGDR with risk of CVD were observed in all subgroups. Significant interactions between eGDR and sex, age and anti-diabetes/hypertension drugs on risk of CVD were also observed (Table 3). In sensitivity analysis 1, we calculated eGDR using BMI instead of waist circumferences. The results based on eGDR_BMI_ were very similar to the results from the main analyses using eGDR based on waist circumference. After multiple adjustment of risk factors, the eGDR_BMI_ was significantly associated with CVD, stroke and cardiac events. Similarly, each 1-SD increase of eGDR_BMI_ was associated with decreased risk of CVD, stroke and cardiac events (Table 4). When we excluded participants with anti-diabetes/hypertension drugs use, significant associations between eGDR and CVD, stroke and cardiac events were also found (Table 4). In sensitivity analysis of longitudinal changes of eGDR on CVD risk, we found a higher decline of eGDR were associated with 19% (*95%CIs,* 11%-23%), 12%(*95%CIs*, 3%-19%) and 16%(*95%CIs,* 11%-20%) risk of CVD, stroke and cardiac events, respectively (Supplemental Table 2).

**Table 3:** Subgroup analysis of the association between eGDR and risk of CVD

Characteristics	eGDR(mg/kg/min)				*P* value	*P-interaction*
	< 8.92	8.92–10.41	10.41–11.26	≥ 11.26		
Sex						
Male	1.00(Ref)	0.91(0.74–1.13)	0.70(0.57–0.87)	0.59(0.47–0.74)	< 0.001	0.005
Female	1.00(Ref)	0.98(0.83–1.16)	0.67(0.55–0.82)	0.57(0.46–0.70)	< 0.001	
Diabetes						
No	1.00(Ref)	0.98(0.85–1.13)	0.70(0.60–0.81)	0.59(0.50–0.69)	< 0.001	0.110
Yes	1.00(Ref)	0.97(0.45–2.09)	0.89(0.50–1.58)	0.75(0.56–0.96)	0.003	
Age, years						
< 60	1.00(Ref)	0.91(0.76–1.10)	0.64(0.52–0.78)	0.55(0.44–0.68)	< 0.001	< 0.001
≥ 60	1.00(Ref)	1.04(0.87–1.25)	0.78(0.63–0.95)	0.65(0.53–0.81)	< 0.001	
Place of residence						
Urban	1.00(Ref)	0.87(0.74–1.04)	0.66(0.55–0.78)	0.57(0.47–0.68)	< 0.001	0.681
Rural	1.00(Ref)	1.10(0.89–1.36)	0.73(0.56–0.93)	0.57(0.43–0.76)	< 0.001	
Smoking						
No	1.00(Ref)	0.97(0.82–1.14)	0.68(0.57–0.82)	0.59(0.48–0.72)	< 0.001	0.465
Yes	1.00(Ref)	0.93(0.75–1.16)	0.69(0.55–0.87)	0.57(0.45–0.72)	< 0.001	
Drinking						
No	1.00(Ref)	0.99(0.84–1.17)	0.70(0.58–0.84)	0.61(0.50–0.74)	< 0.001	0.954
Yes	1.00(Ref)	0.90(0.72–1.11)	0.68(0.54–0.85)	0.54(0.42–0.69)	< 0.001	
Education level, n (%)						
Below primary school	1.00(Ref)	0.98(0.78–1.23)	0.67(0.52–0.87)	0.60(0.46–0.78)	< 0.001	0.114
Primary school	1.00(Ref)	0.98(0.79–1.21)	0.73(0.58–0.91)	0.55(0.43–0.71)	< 0.001	
Middle school	1.00(Ref)	1.07(0.78–1.47)	0.72(0.51–1.02)	0.69(0.47–0.98)	0.022	
High school or above	1.00(Ref)	0.73(0.49–1.09)	0.67(0.43–1.04)	0.52(0.32–0.84)	0.002	
Anti-diabetes/hypertension drugs						
No	1.00(Ref)	0.91(0.79–1.05)	0.66(0.57–0.77)	0.56(0.47–0.65)	< 0.001	0.002
Yes	1.00(Ref)	1.02(0.66–1.29)	0.94(0.56–1.09)	0.58(0.43–0.77)	0.005	

In the multivariate models, confounding factors such age, sex, place of residence, education level, blood glucose, smoking, drinking, systolic blood pressure, physical activity, chronic diseases (dyslipidemia, chronic lung disease) and medications (anti-hypertensive and anti-dyslipidemic) were included unless the variable was used as a subgroup variable

**Table 4:** Sensitivity analysis of the association between eGDR_BMI_ and risk of CVD, stroke and cardiac events

Characteristics	eGDR(mg/kg/min)				*P* trend	Each SD increase^a^
	< 8.99	8.99–10.52	10.52–11.22	≥ 11.22		
**Sensitivity analysis 1**						
**CVD**						
Multivariable-adjusted^b^	1.00(Ref)	0.95(0.83–1.08)	0.73(0.63–0.84)	0.59(0.51–0.69)	< 0.001	0.84(0.80–0.88)
**Stroke**						
Multivariable-adjusted^b^	1.00(Ref)	0.99(0.79–1.25)	0.85(0.63–1.15)	0.84(0.65–0.98)	0.009	0.91(0.85–0.99)
**Cardiac events**						
Multivariable-adjusted^b^	1.00(Ref)	0.96(0.83–1.12)	0.71(0.61–0.84)	0.55(0.46–0.66)	< 0.001	0.84(0.79–0.88)
**Sensitivity analysis 2**						
**CVD**						
Multivariable-adjusted^b^	1.00(Ref)	0.91(0.79–1.05)	0.66(0.57–0.77)	0.56(0.47–0.65)	< 0.001	0.82(0.77–0.86)
**Stroke**						
Multivariable-adjusted^b^	1.00(Ref)	0.92(0.72–1.18)	0.73(0.56–0.97)	0.64(0.47–0.88)	0.003	0.88(0.79–0.98)
**Cardiac events**						
Multivariable-adjusted^b^	1.00(Ref)	0.92(0.78–1.09)	0.66(0.55–0.79)	0.55(0.46–0.67)	< 0.001	0.82(0.77–0.87)

In sensitivity analysis 1, the eGDR_BMI_ was calculated based on BMI. In sensitivity analysis 2, those with anti-diabetes/hypertension drugs use were excluded

*CVD* Cardiovascular diseases, *eGDR* Estimated glucose disposal rate

^a^Each SD increase in eGDR (1.71 mg/kg/min)

^b^Multivariable-adjusted for age, sex, place of residence, education level, blood glucose, smoking, drinking, systolic blood pressure, physical activity, chronic diseases (dyslipidemia, chronic lung disease) and medications (anti-hypertensive and anti-dyslipidemic)

## Discussion

Our nationwide study provided population-based novel evidence of eGDR and CVD in general population. Results suggested that eGDR was positively correlated with the occurrence of CVD, stroke and cardiac events. We found higher eGDR (decreased insulin resistance) was associated with lower risk of CVD, stroke and cardiac events, and there were liner relationships. In addition, subgroup and sensitivity analysis supported the stable association between eGDR and CVD, stroke and cardiac events. Furthermore, a higher decline of eGDR were associated with increased risk of CVD, stroke and cardiac events. Our findings supported the hypothesis that eGDR may be an important predictor of CVD, and potential target for CVD prevention in general population.

Insulin resistance is of utmost importance as an underlying mechanism for increased risk of CVD. Systemic insulin resistance produces atherogenic lipid phenotypes by increasing very low-density lipoprotein particles, which are metabolized into residual lipoproteins that promote atherosclerosis. Pro-inflammatory and procoagulant states caused by insulin resistance also play an important role in the formation of atherosclerosis [7]. Furthermore, insulin resistance was significantly correlated with vascular function, hypertension and macrophage accumulation [5]. Thus, evaluation of insulin resistance has emerged as an additional and significant CVD risk factor in the assessment of chronic vascular complications and mortality in general population and individuals with specific diseases [24–26]. Consideration the invasiveness and expenditure of traditional techniques in large-scale population studies, the eGDR was developed and proven to have a high precision of insulin resistance.

Since the eGDR was released, the score has been used in several investigations for the assessment of clinical diabetic chronic complications in both the T1D and T2D population [5]. In a study of 191 T1D participants with no prior CVD, the eGDR was independently related to ≥ 2 plaques (*P* = 0.018) and maximum plaque height (*P* < 0.01), suggesting that eGDR may be a predictor of preclinical carotid atherosclerosis for CVD risk [27]. In another study of 2,151 T1D participants, results indicated that eGDR was strongly associated with the presence of both microvascular and macrovascular complications than BMI, and the relationship was independent of HbA1c [28]. When it comes to T2D, Zabala et al. demonstrated that a higher eGDR was associated with a decreased risk of stroke (HRs = 0.60: 0.48–0.76) and death (HRs = 0.60: 0.48–0.76) in individuals with T2D. In addition, no statistically significantly different from the participants with or without insulin treatment [13]. In present study, our extended the current literature of eGDR on CVD in general population by showing positive associations of eGDR and CVD, stroke and cardiac events. Furthermore, we found significant interactions between eGDR and sex, age and anti-diabetes/hypertension drugs on risk of CVD. However, significant associations between eGDR with risk of CVD were observed in all subgroups, which was consistent with previous reported studies [29–31]. The present study provides a more valid appraisal of the relationship between eGDR and CVD, stroke and cardiac events.

Obesity was a well-known risk factor for multiple disease conditions including CVD and stroke [32]. In the eGDR formula, when waist circumference was used in place of waist-to-hip ratio, a similar pattern of results was obtained. In current study, after replacement the BMI in the eGDR formula, instead of waist circumference, there were robust association between eGDR_BMI_ and CVD risk. Most former publications reported various complications in both T1D and T2D patients. In our subgroup analysis according to diabetes, it is noteworthy that the significant associations were found in participants with or without diabetes. Therefore, IR indicators (assessed by eGDR) seem to be not merely associated with complications in diabetes patients, but also has a certain predictive value in people without diabetes. According to previous reports, in the eGDR formula, the explained highest attributable relative risk for CVD was hypertension, followed by BMI, HbA1c, and waist circumference, all of which are well-known risk factors for CVD [13, 33]. Thus, the potential mechanisms under eGDR on CVD may indirect mediate thorough its effect on other variables such as blood pressure, lipidemia and glycaemia. Future well-designed experimental research and prospective clinical studies are certainly warranted to clarify the potential biological mechanisms. In addition, future studies are required to certify the optimal cut-off of eGDR that been applicable for implemented in routine clinical practice, and to formulate relevant management strategies of eGDR in both diabetes and non-diabetes patients.

The present study was based on the data from the CHARLS study, which is a large nationally representative cohort study with a high response rate, and potential confounders were collected and controlled in the multivariable models. Our findings provided extra evidence of eGDR in clinical application that incorporating eGDR into routine practice may help healthcare professionals and patients appreciate the importance of risk factors other than glucose levels, potentially improving long-term outcome in people with or without diabetes, particularly those whom are insulin resistant. However, there some limitations. First, the eGDR is a measure of insulin resistance developed for individuals with T1D, although the correlation between euglycaemic hyperinsulinaemic clamp and eGDR were good. We cannot conclude that eGDR can be replaced with the gold standard clamp technique. Second, the study outcome of CVD was based on self-reported doctor’s diagnosis of stroke or cardiac events, which may cause information bias. However, self-reported history of disease has been proven to possess relatively good reliability [34]. Third, there was significant difference between included and excluded groups in most of the baseline characteristics, which may reduce the credibility of the results. Finally, the present study was not a prespecified analysis. This observational analysis could be influenced by potential biases and confounding factors. Therefore, our study may only generate hypotheses for future studies.

In conclusion, our findings indicated that individuals with a low eGDR (a simple measure of insulin resistance), was associated with an increased risk of CVD, stroke and cardiac events in general Chinese population. Consequently, eGDR could emerge as a chief non-glycaemic variable for the early detection of individual with high risk of CVD, stroke and cardiac events in general Chinese population.

## Supplementary Information

**Additional file 1: Supplemental Table 1.** Baseline characteristics between the included and excluded groups. **Supplemental Table 2.** Association between eGDR change and risks for CVD, stroke and cardiac events (*N*=4075)

## Data Availability

The details of the CHARLS data are available at its website (http://charls.pku.edu.cn/en).

## Abbreviations

eGDR: Estimated glucose disposal rate
CVD: Cardiovascular disease
HOMA-IR: Homeostasis model assessment of insulin resistance
HbA1c: Glycosylated hemoglobin A1c
CHARLS: China Health and Retirement Longitudinal Study
FPG: Fasting plasma glucose
BMI: Body mass index
