# Supplementary material for: Estimated glucose disposal rate and risk of cardiovascular disease: evidence from the China Health and Retirement Longitudinal Study
Source: BMC Geriatr. 2022 Dec 15;22:968. doi: 10.1186/s12877-022-03689-x (PMC9753298; doi:10.1186/s12877-022-03689-x)
Supplement: Supplementary file 1 — Additional file 1: Supplemental Table 1. Baseline characteristics between the included and excluded groups. Supplemental Table 2. Association between eGDR change and risks for CVD, stroke and cardiac events (N=4075) [file 12877_2022_3689_MOESM1_ESM.docx]

**Supplemental Table 1.** Baseline characteristics between the included and excluded groups.

| Characteristics | Included |  | Excluded |  | *P* value |
| --- | --- | --- | --- | --- | --- |
| No. of subjects | 8267 |  | 8957 |  |  |
| Age, years | 58.90±9.40 |  | 59.36±10.32 |  | 0.002 |
| Sex, n (%) |  |  |  |  |  |
| Male | 3916(47.37) |  | 4481(50.03) |  | <0.001 |
| Female | 4351(52.63) |  | 4476(49.97) |  |  |
| Living place, n (%) |  |  |  |  |  |
| Urban | 2762(33.41) |  | 4205(46.95) |  | <0.001 |
| Rural | 5505(66.59) |  | 4752(53.05) |  |  |
| Education level, n (%) |  |  |  |  |  |
| Illiteracy | 2508(30.34) |  | 2260(25.23) |  | <0.001 |
| Primary school | 3362(40.67) |  | 3346(37.36) |  |  |
| Middle school | 1616(19.55) |  | 1936(21.61) |  |  |
| High school or above | 781(9.45) |  | 1415(15.80) |  |  |
| Dyslipidemia, n (%) | 623(7.54) |  | 1051(11.73) |  | <0.001 |
| Smoking, n (%) | 3286(39.75) |  | 3581(39.98) |  | 0.311 |
| Drinking, n (%) | 3277(39.64) |  | 3456(39.16) |  | 0.519 |
| Blood glucose, mg/dl | 109.91±36.92 |  | 111.87±38.33 |  | 0.011 |
| BMI (kg/m^2^) | 23.05(20.79-25.54) |  | 23.20(20.78-25.90) |  | 0.002 |
| SBP, mmHg | 129.87±21.16 |  | 136.02±19.31 |  | <0.001 |
| DBP, mmHg | 75.60±11.60 |  | 77.43±12.76 |  | <0.001 |

BMI: body mass index; SBP: systolic blood pressure; DBP: diastolic blood pressure;

Continuous variables are expressed as mean± standard deviation, or as median (interquartile range). Categorical variables are expressed as frequency (percent).

**Supplemental Table 2.** Association between eGDR change and risks for CVD, stroke and cardiac events (N=4075).

| Characteristics | eGDR change (mg/kg/min) | | | | *P* trend |
| --- | --- | --- | --- | --- | --- |
|  | <0.11 | 0.11-0.57 | 0.57-1.10 | ≥1.10 |  |
| **CVD** | | | | | |
| Case, n(%) | 72(7.06) | 98(9.63) | 135(13.25) | 143(14.05) |  |
| Unadjusted | 1.00(Ref) | 1.37(1.01-1.86) | 1.93(1.45-2.57) | 2.06(1.55-2.73) | <0.001 |
| Age and sex- adjusted | 1.00(Ref) | 1.38(1.02-1.88) | 1.90(1.43-2.53) | 1.98(1.49-2.62) | <0.001 |
| Multivariable-adjusted^*^ | 1.00(Ref) | 1.33(0.98-1.81) | 1.77(1.33-2.37) | 1.85(1.39-2.47) | <0.001 |
| **Stroke** |  |  |  |  |  |
| Case, n(%) | 28(2.75) | 43(4.22) | 59(5.79) | 57(5.60) |  |
| Unadjusted | 1.00(Ref) | 1.55(0.96-2.49) | 2.14(1.36-3.35) | 2.07(1.32-3.25) | <0.001 |
| Age and sex- adjusted | 1.00(Ref) | 1.62(1.00-2.60) | 1.99(1.27-3.31) | 2.11(1.35-3.31) | 0.002 |
| Multivariable-adjusted^*^ | 1.00(Ref) | 1.34(0.83-2.17) | 1.43(0.89-2.31) | 1.64(1.03-2.61) | 0.004 |
| **Cardiac events** |  |  |  |  |  |
| Case, n(%) | 49(4.80) | 60(5.89) | 86(8.44) | 92(9.04) |  |
| Unadjusted | 1.00(Ref) | 1.23(0.84-1.79) | 1.79(1.26-2.54) | 1.92(1.36-2.71) | <0.001 |
| Age and sex- adjusted | 1.00(Ref) | 1.21(0.83-1.77) | 1.75(1.23-2.49) | 1.83(1.29-2.59) | <0.001 |
| Multivariable-adjusted^*^ | 1.00(Ref) | 1.26(0.86-1.84) | 1.77(1.25-2.52) | 1.88(1.33-2.68) | <0.001 |

CVD: Cardiovascular diseases; eGDR: estimated glucose disposal rate

^*^Multivariable-adjusted for age, sex, place of residence, education level, blood glucose, smoking, drinking, systolic blood pressure, physical activity, chronic diseases (dyslipidemia, chronic lung disease) and medications (anti-hypertensive and anti-dyslipidemic).
